# Editorial: Molecules, environments, and neurological disorders

**DOI:** 10.3389/fendo.2023.1244800

**Published:** 2023-08-28

**Authors:** Cai Qi, Lu Wang, Yong Lu, Chunyu Jin

**Affiliations:** ^1^ Department of Neuroscience, Yale School of Medicine, New Haven, CT, United States; ^2^ Department of Neurosciences, School of Medicine, University of California, San Diego, La Jolla, CA, United States; ^3^ Department of Radiology, Ruijin Hospital, Shanghai Jiao Tong University School of Medicine, Shanghai, China; ^4^ Division of Endocrinology and Metabolism, School of Medicine, University of California, San Diego, La Jolla, CA, United States

**Keywords:** environment, molecules, genetics, epigenetics, neurological disorders

Both nature and nurture play significant roles in determining the likelihood of normal or abnormal development of the individuals. Recent clinical genetic studies have identified numerous variants associated with neurological disorders, providing potential genetic causes which can be further explored to understand the underlying pathological mechanisms. Additionally, twin studies in 1999 highlighted the contribution of non-genetic factors in development of neurological disorders (1, 2). Indeed, the genetic variants and genomic homeostasis can be perturbed by environmental molecules, eliciting diseases pathogenesis with few changes in the genome (3). For example, there is compelling evidence from rodents that metabolic changes induced by nutrition intake in parentals has long-lasting effects on developmental and behavioral phenotypes of their offsprings (4–7). Therefore, gaining a better understanding of the interactions between environmental molecules and genetic/genomic factors will benefit both preventive medicine and patients by correcting the abnormal processes without putting the integrity of genome at risk.

The brain receives information from external stimulation as well as internal states and thus is affected by environments heavily. Under most circumstances, environmental stimulation causes neurological phenotypes *via* gene expression by acting on the epigenome. The environmental factors include chemicals (8), hormones (9), and neurotransmitters. At the embryonic stage, the maternal environment is crucial for the proper development of the nervous system of the progenies. Zika causes microcephaly after maternal infection (10, 11). It has also been reported that exposure of pollutant induced maternal immune response which will undermine the microglia function and synaptic development (12). In a mini-review, Doi et al. summarized the latest studies on maternal immune activation and drug usage on the development of psychiatric disorders such as autism spectrum disorder, and schizophrenia, with a focus on synapse development related molecules, neurotransmitter, and hormone changes. Endocrine-disrupting compounds have been reported to elicit diseases through epigenetic modifications (13). In a research article by Martinez et al., they reported that embryonic exposure to thyroid hormone causes autism spectrum-like behavior in the third-generation wild-type mouse through epigenetic modification of autistic genes. Their study revealed how environmental exposure was “recorded” and passed through generations. With the advances of sequencing technology and the lowering cost, more and more disorders will be dissected at an otherwise unattainable resolution. Recently, we have started to appreciate the contribution of more and more variants in probands of various disorders. Among these variants, a proportion are *de novo*. Genetic analysis will enable us to uncover when and where these variants were generated in the lineage. In this Research Topic, Vado et al. reported the distribution of variants in families with inactivating PTH/PTHrP signaling disorder type2. Zhang et al. reported a novel mutation in the G*LI2* gene and reviewed other causative mutations. The accumulation of such kind of knowledge will accelerate the application of precise medicine as suggested by Gilis-Januszewska et al. who used Cushing syndrome as an example. We are expecting continued progress in this field and looking forward to the clinical applications.

## Author contributions

CQ, LW, and CJ write the manuscript. All authors consent on the contents of the manuscript. All authors contributed to the article and approved the submitted version.
